# The current approach to initial crystallization screening of proteins is under-sampled

**DOI:** 10.1107/S0021889813008030

**Published:** 2013-04-18

**Authors:** Fabrice Gorrec

**Affiliations:** a MRC Laboratory of Molecular Biology, Francis Crick Avenue, Cambridge Biomedical Campus, Cambridge CB2 0QH, England

**Keywords:** Macromolecular crystallization, screen formulation, crystallization reagents, crystal structure

## Abstract

Protein crystallization conditions that resulted in crystal structures published by scientists at the MRC Laboratory of Molecular Biology (MRC-LMB, Cambridge, UK) have been analysed. It was observed that the more often a crystallization reagent had been used to formulate the initial conditions, the more often it was found in the reported conditions that yielded diffraction quality crystals. The present analysis shows that, despite the broad variety of reagents, they have the same impact overall on the yield of crystal structures. More interestingly, the correlation implies that, although the initial crystallization screen may be considered very large, it is an under-sampled combinatorial approach.

## Abstract 2

Forty-seven main reagents included in a large set of macromolecular crystallization conditions are shown to have a similar impact overall on the yield of crystal structures. It is also shown that the conditions formulated with such reagents are under-sampled.

## 1. Abbreviations

HEPES: 4-(2-hydroxyethyl)piperazine-1-ethanesulfonic acid

DNA: deoxyribonucleic acid

MES: 2-(*N*-morpholino)ethanesulfonic acid

MRC-LMB: Medical Research Council Laboratory of Molecular Biology

MPD: 2-methyl-2,4-pentanediol

OAc: acetate

PEG: polyethylene glycol

RNA: ribonucleic acid

Tris: 2-amino-2-hydroxymethyl-propane-1,3-diol

## 2. Introduction

Macromolecular crystallization is often referred to as the main bottleneck for structure determination by X-ray crystallography because of the low yield of well diffracting crystals that is obtained for any given sample (Chayen & Saridakis, 2008 ▶). As a consequence, samples are tested for crystallization against a variety of aqueous solutions that contain reagents promoting crystallization (termed ‘conditions’). Since hundreds of crystallization reagents can be employed, a huge matrix of possible reagent combinations should ideally be investigated to initiate even the least probable crystal nucleation and growth. However, the amount of sample available is generally the limiting factor, and hence routine initial crystallization screens have been formulated with a limited number of suitable conditions selected empirically (‘sparse-matrix screens’) (Jancarik & Kim, 1991 ▶). Alternatively, initial conditions have been formulated systematically (with suitable reagents also selected empirically) to yield ‘incomplete factorial screens’ (Carter & Carter, 1979 ▶) or ‘grid screens’. Reagents employed to formulate initial conditions are commonly divided into three different categories, comprising precipitating agents, buffers and additives. Precipitating agents are used at relatively high concentrations, while buffers and additives are usually used at lower concentrations, but in practice the division between these three categories is often blurred. For example, polycarboxylic acids act as precipitating agents (McPherson, 2001 ▶), but they are also useful additives for protein stabilization (Maclean *et al.*, 2002 ▶), as well as functioning as buffers. In addition, after initial crystal hits have been identified, subsequent optimization experiments are typically required to obtain diffraction quality crystals, and hence the published final condition formulations often differ from those employed initially. Consequently, we decided to analyse our crystallization data using the final optimized formulations without categorizing the reagents into buffers, precipitants or additives.

An analysis was performed of the published conditions for crystal growth that resulted in protein structures, determined over a period of seven years. This included the conditions from 94 structures determined by 15 groups who use the crystallization facility at the LMB. Studying the yield of crystal structures in relation to the crystallization conditions, as opposed to the yield of crystals observed in initial crystallization screens, allowed us to bypass any biases in the reporting of ‘crystallization’, such as (for example) the personal interpretation of crystallization experiments, by recording only the presence or absence of crystals observed in trays. Furthermore, it also allowed us to include all parameters that lead to diffraction quality crystals for structure determination, as they can be altered by the formulation of the conditions. For example, glycerol might help with crystallization and protein stability, but also makes it easier to cryo-cool the resulting crystals and hence increases the probability of obtaining a structure.

**Figure 1:**
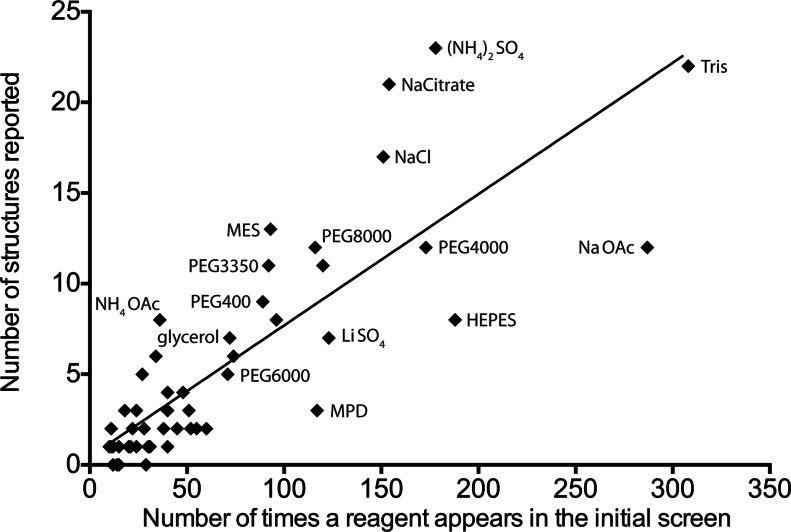
Correlation between the number of times a crystallization reagent is present in the initial crystallization screen at the LMB and the number of times that particular reagent appeared in the optimized crystallization conditions used to determine the resulting structures (*i.e.* the number of structures reported). Among the 55 main reagents represented, we highlight eight examples that fit the correlation well (five PEGs, plus glycerol, LiSO_4_ and Tris) and eight outliers [NaOAc, HEPES, MPD, (NH_4_)_2_SO_4_, NaCitrate, NaCl, MES and NH_4_OAc].

Strikingly, there is a strong correlation between the number of occurrences of the reagents in our large set of initial conditions and their occurrence across the reported conditions for published structures. In other words, overall, the more often a reagent has been employed to formulate the initial screen, the more often it is found in the final conditions used to determine crystal structures. At first, this outcome may sound obvious because these reagents were empirically selected. Nevertheless, this correlation was possible only if the different reagents employed in commercial screens had a potentially similar impact on the process. More interestingly, another interpretation of this outcome is the under-sampling of suitable reagents: since the overall impact, and not the occurrence, is similar, no reagent is found too many times in the screen (althougth note that this is less true for three outliers which clearly have low impact: MPD, HEPES and sodium acetate; see Fig. 1 ▶). We therefore argue that, by adding more suitable conditions (and reagents) to the screen, even more structures will be determined.

## 3. Materials and methods

All protein samples were screened initially using between 1152 and 1440 crystallization conditions chosen from various commercially available or commonly used sparse matrices, incomplete factorial screens and grid screens (Table S1 in the supplementary materials^1^). Between February 2002 and April 2009, more than four million individual crystallization experiments were set up using the vapour-diffusion sitting-drop technique, with a final drop size of 100–500 nl (Stock *et al.*, 2005 ▶). In total, 94 unique published protein crystal structures were determined by X-ray diffraction at the LMB using various techniques and methods (Table S2). Among the samples tested were proteins involved in a wide variety of cellular processes, such as the bacterial cytoskeleton (Low & Löwe, 2006 ▶), phos­phoino­sitide signalling (Teo *et al.*, 2006 ▶), intracellular immunity (James *et al.*, 2003 ▶), Wnt signal transduction (Fiedler *et al.*, 2008 ▶), nuclear trafficking (Lee *et al.*, 2005 ▶) and the sculpting of cell membranes (Ford *et al.*, 2002 ▶). Published results with transmembrane protein samples were excluded from this study since they generally require different approaches (Warne *et al.*, 2009 ▶). Also excluded were samples containing long nucleic acids (RNA or DNA), since they have different physico-chemical properties. Several crystal structures resulting from the same project were considered non-redundant if the corresponding protein(s) had a difference of ±5% in molecular weight or if a different crystal space group was later observed. The results include 30 hetero-oligomeric complexes. The average mol­ecular weight of the crystallized proteins was 37 kDa, with 18 samples having molecular weights above 50 kDa. Note that three structures are complexes containing short stretches of DNA (PDB codes 2ve9, 1w0t and 1w0u; Table S2).

Our data set consists of 106 optimized conditions found in publications for the 94 unique structures solved at the MRC-LMB (Table S2). An additional 12 conditions are included because, in some cases, two crystallization conditions were reported for various reasons (*e.g.* crystallization of selenomethionine-substituted samples). Reagents found less than ten times in the 1440 initial conditions were excluded, in order to avoid positively biasing the correlation observed in Fig. 1 ▶ with a large number of data points near the origin. The final number of reagents taken into account was 55 (Table S3; the *x* value represents the number of times a reagent appears in our initial screen and the *y*_obs_ value represents the number of structures reported). Table S3 shows deviation values |∊| in red font when *y*_obs_ are located more than one and a half times the standard deviation above or below the best fit line (*i.e.* |∊| > 1.5δ).

## 4. Results and discussion

Fig. 1 ▶ shows the number of occurrences of the 55 main reagents in our initial screen, plotted against the occurrence within all reported conditions for the published structures. When these data are fitted by linear least squares, we obtain a significant correlation of *R* ^2^ = 0.69. This suggests that, overall, the reagents were well suited and they had a similar impact on the yield of crystal structures. The group of PEGs, for example, fits the observed correlation particularly well (*i.e.* the corresponding data points in Fig. 1 ▶ do not deviate from the best fit line). PEGs appear in 809 conditions of our initial screen (Table S3). Pragmatically, such reagents are highly represented not only because they are suitable for crystallization (McPherson, 1976 ▶) but also because they are stable and relatively easy to handle; in addition, they do not alter the pH of the conditions (a main parameter for crystallization). Finally, it is important to note that they are cost effective. Cost effectiveness must also be the reason why the buffer Tris is the most highly represented reagent (308 times), since it does not have an exceptional impact (note that high impact is observed for five outliers: ammonium sulfate, sodium citrate, sodium chloride, MES and ammonium acetate; see Fig. 1 ▶).

It was observed that there are eight obvious outliers that contradict the general trend and hence, strictly speaking, the number of reagents exhibiting a similar impact is 47 (Table S3). For example, sodium chloride is not thought to alter solubility as effectively as ammonium sulfate (Arakawa & Timasheff, 1985 ▶), although here it appears to be a highly effective crystallization reagent. A possible explanation might be a preference of proteins for sodium chloride because of its prevalence in the environment. The low impact of MPD may be explained by the particular way this reagent alters the equilibrium of vapour diffusion (Kimber *et al.*, 2003 ▶). However, one might ask why do sodium citrate, ammonium acetate and sodium acetate exhibit such different efficiencies despite being from the same family of reagents? Also, what is the explanation for the low suitability of HEPES buffer compared with MES? Unfortunately, we are looking at a global statistic and therefore cannot explain the nature of the impact of particular reagents (or groups of reagents) in terms of their physico-chemical properties and how they specifically alter our crystallization efforts. For that, we would need a much larger data set, and a thorough analysis of various biases associated with crystallization experiments and reagents (Wooh *et al.*, 2003 ▶; Newman *et al.*, 2007 ▶; St John *et al.*, 2008 ▶).

A previously published analysis of a much larger data set, although based on the yield of crystals rather than on the final conditions, showed that protein crystallization will most likely occur with an initial screen limited to 48 conditions (Kimber *et al.*, 2003 ▶). This implied that a small screen may be used initially. The basis for the observations of Kimber *et al.* appears to be related to the nature of the protein being crystallized in terms of, for example, size and stability. If the reagents of our large initial screen were over-sampled, the highly represented group of reagents would generally exhibit a lower impact and the plot in Fig. 1 ▶ would reach a plateau. We do not observe such a plateau. This suggests that our initial crystallization screen is more likely to reflect an under-sampled combinatorial approach. The underlying reason is that the combinations of reagents employed alter the combinations of variables associated with the main parameters of crystal structure determination. These parameters are numerous. They are related to the nature of the protein, the nature of the experiment, the possibilities of protein–protein interactions, the type of crystals obtained, the reaction of the crystals to freezing *etc*. Hence, there are an enormous number of subsequent combinations of variables that can be tested during protein crystallization. Pragmatically, however, there is only a limited supply of purified protein and finite time and resources to set up and analyse crystallization trials. Clearly, it is important to maximize the best chances of obtaining crystals, so over-sampling is undesirable as it would be wasteful. The analysis presented here demonstrates that, although the initial crystallization screen at the MRC-LMB may be considered very large, in actual fact it is still under-sampled, and therefore a more successful screen can only be obtained with more combinations of reagents. Additional conditions should not only optimize the use of the commonly employed suitable reagents, but also include more reagents. Ultimately, additional conditions should not imply more effort and cost in the process: the development of technology and technique should facilitate more extensive screening.

## 5. Conclusion

This study provides an illustration of the complexity of crystal structure determination and explains why the development of more efficient crystallization screens is still challenging (McPherson & Cudney, 2006 ▶). We are constantly testing new conditions and will eventually integrate them into our initial screen (Gorrec, 2009 ▶; Gorrec *et al.*, 2011 ▶). It will be interesting to repeat the analysis presented here when a further 100 crystal structures have been obtained to see if the crystallization space is then sampled more thoroughly, ultimately up to saturation. Technological developments should facilitate the use of much larger initial screens, maximizing the chance of obtaining crystals with the minimum of resources. These developments imply a continuous miniaturization of crystallization experiments, as seen with microfluidic (Hansen *et al.*, 2002 ▶) and acoustic (Villaseñor *et al.*, 2012 ▶) technologies. They also imply a higher throughput for the screening of crystals, as seen with diffraction through crystal drops (Jacquamet *et al.*, 2004 ▶) and liquid jets (Boutet *et al.*, 2012 ▶).

## Supplementary Material

Table S1: Formulation of our initial screen. DOI: 10.1107/S0021889813008030/he5578sup1.xls

Table S1: Formulation of our initial screen. DOI: 10.1107/S0021889813008030/he5578sup4.ods

Table S2: Our data set (106 conditions for 94 unique crystal structures). DOI: 10.1107/S0021889813008030/he5578sup2.xls

Table S2: Our data set (106 conditions for 94 unique crystal structures). DOI: 10.1107/S0021889813008030/he5578sup5.ods

Table S3: data for Figure 1. DOI: 10.1107/S0021889813008030/he5578sup3.xls

Table S3: data for Figure 1. DOI: 10.1107/S0021889813008030/he5578sup6.ods

## Footnotes

1 Extra tables discussed in this paper are available from the IUCr electronic archives (Reference: HE5578). Services for accessing these data are described at the back of the journal.
